# Response of Maize Varieties (*Zea mays* L.) to the Application of Classic and Stabilized Nitrogen Fertilizers—Nitrogen as a Predictor of Generative Yield

**DOI:** 10.3390/plants12030600

**Published:** 2023-01-29

**Authors:** Piotr Szulc, Daniel Krauklis, Katarzyna Ambroży-Deręgowska, Barbara Wróbel, Gniewko Niedbała, Mohsen Niazian, Marek Selwet

**Affiliations:** 1 Department of Agronomy, Poznań University of Life Sciences, Dojazd 11, 60-632 Poznań, Poland; 2 Experimental Station for the Cultivar Testing in Chrząstowo, Research Centre for Cultivar Testing in Słupia Wielka, Chrząstowo 8, 89-100 Nakło nad Notecią, Poland; 3 Department of Mathematical and Statistical Methods, Poznań University of Life Sciences, Wojska Polskiego 28, 60-637 Poznań, Poland; 4 Institute of Technology and Life Sciences-National Research Institute, 3 Hrabska Avenue, 05-090 Raszyn, Poland; 5 Department of Biosystems Engineering, Faculty of Environmental and Mechanical Engineering, Poznań University of Life Sciences, Wojska Polskiego 50, 60-627 Poznań, Poland; 6 Field and Horticultural Crops Research Department, Kurdistan Agricultural and Natural Resources Research and Education Center, Agricultural Research, Education and Extension Organization (AREEO), Sanandaj 6616936311, Iran; 7 Department of Soil Science and Microbiology, Poznań University of Life Sciences, Szydłowska 50, 60-656 Poznań, Poland

**Keywords:** maize, nitrogen, inhibitor, stabilization, grain yield, N application effectiveness factors

## Abstract

The study presents the results of a 3-year field trial aimed at assessing the yield and efficiency indicators of nitrogen application in the cultivation of three maize cultivars differing in agronomic and genetic profile. The advantages of the UltraGrain stabilo formulation (NBPT and NPPT) over ammonium nitrate and urea are apparent if a maize cultivar capable of efficient nutrient uptake in the pre-flowering period and effective utilization during the grain filling stage is selected. Therefore, the rational fertilization of maize with urea-based nitrogen fertilizer with a urease inhibitor requires the simultaneous selection of cultivars that are physiologically profiled for efficient nitrogen utilization from this form of fertilizer (“stay-green” cultivar). The interaction of a selective cultivar with a high genetically targeted potential for nitrogen uptake from soil, combined with a targeted selection of nitrogen fertilizer, is important not only in terms of production, but also environmental and economic purposes.

## 1. Introduction

**Figure 1:**
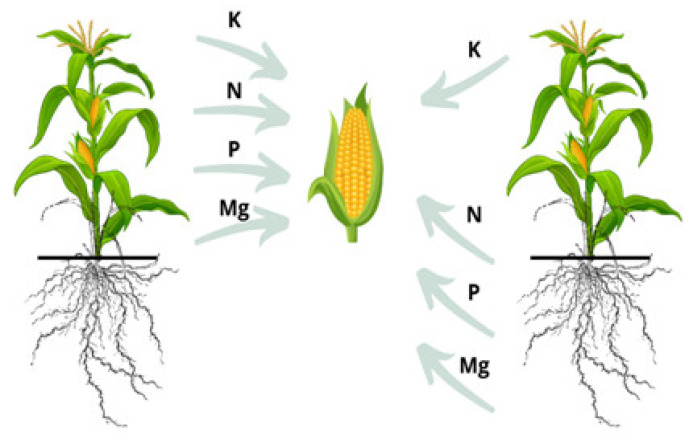
Nutrient remobilization pattern during the grain filling period depending on the type of maize hybrid (on the right, the “stay-green” cultivar, on the left, the traditional cultivar) [13].

Nitrogen supplied to the soil in the form of mineral fertilizers is not 100% utilized by crops [1,2,3]. Its mineral forms are absorbed by plants but also leached from the soil to groundwater, causing its eutrophication [4]. According to Modolo et al. [5], more than 50% of nitrogen fertilizer applied worldwide is not used by plants, and the uptake of this component by maize is approximately 50%. Nitrogen is an essential nutrient for maize and a key determinant (predictor) of grain yield, especially due to its role in photosynthesis and other biological processes [6]. In order to reduce the production of excessive mineral forms of nitrogen in the soil, it is necessary to correctly determine the doses of nitrogen fertilizers, taking into account the physicochemical properties of the soil, the type of nitrogen fertilizer, and the nutritional requirements of plants [7,8]. Achieving an increase in the efficiency of mineral nitrogen fertilizer application is not easy, as plants absorb nitrogen in the form of nitrate or ammonium ions through their roots from the soil solution [9]. In agricultural practice, the doses of mineral fertilizers, including nitrogen fertilizers, are determined according to the plant’s nutritional demand without taking into account the abundance of assimilable nutrients in the soil [1,10,11]. This results in the formation of an excess of soluble fertilizing components in the soil and their increased leaching, which, on the one hand, places a burden on the environment, and on the other, reduces the effectiveness of the component application [12]. Hence, rationalizing the use of nitrogen fertilizer in maize cultivation is an important issue for sustainable agriculture, as it can reduce the negative impacts on the surrounding environment. There are works in the literature on maize fertilization involving various forms of nitrogen fertilizers; however, there are no studies comparing the responses of individual types of maize cultivars with various forms of nitrogen fertilizers [1]. This is very important from a scientific point of view because, as shown by Szulc et al. [13], the “stay-green” maize hybrid is characterized by a negative coefficient of nitrogen remobilization, i.e., soil resources are the decisive source of this nutrient in the period of generative crop formation (Figure 1). Therefore, in the cultivation of such cultivars, only slow-acting nitrogen fertilizers (e.g., urea) should be used, as they are best suited to the rhythm of their growth. 

Stabilized nitrogen fertilizers contain nitrification or urease inhibitors. The main purpose of their use is to increase the efficiency of plant fertilization with nitrogen (by reducing the number of applications and ensuring a wider range of application dates), as well as improve environmental conditions by reducing the risk of releasing excess nitrogen into the groundwater and air [14]. Nitrification inhibitors reduce the intensity of the microbial transformations of ammonium ions (${\mathrm{NH}}_{4}^{+}$) towards nitrate ions (${\mathrm{NO}}_{3}^{-}$) and free nitrogen (gaseous N_2_ and nitrous oxide N_2_O—greenhouse gases) in the soil. A urease inhibitor, on the other hand, is a compound that temporarily slows down the enzymatic conversion of urea to CO_2_ and NH_3_ due to its inhibitory effect on urease. This is of great importance, especially in alkaline soils, as it reduces nitrogen losses due to ammonia volatilization. A urease inhibitor blocks the conversion of urea to ammonia for one to two weeks. This reduces the risk of losses due to its evaporation, providing a major advantage over non-stabilized urea [2,15]. Data from the literature provide different information on the response of maize to nitrogen application [16,17]. According to some authors, very high doses of nitrogen increased yield [18]. The expected maize response to increasing levels of nitrogen fertilization is referred to as a “creeping reaction”, i.e., even high and very high doses of the component cause small increases in yield or at least do not cause its reduction. The results of other authors [19] indicated a high reduction in grain yield under the influence of a certain dose of nitrogen, or the lack of response to this component. This raises the basic question of how to control nitrogen metabolism in maize. In agricultural practice, the implementation of this objective is based on the following activities: (i) the control of nitrogen fertilization dose [11], (ii) control of nitrogen metabolism by introducing nitrogen-balancing minerals [16], and (iii) selection of nitrogen carrier [20]. From 1st August 2021, the use of granular urea is prohibited, and only granular urea containing either a urease inhibitor or a biodegradable coating is allowed. On the basis of the current knowledge on the response of maize to the application of nitrogen fertilizers, the working hypothesis of the study was as follows: classical and stabilized nitrogen fertilizers would affect the yield of maize cultivars and N utilization from the mineral fertilizer dose. These issues were therefore the focus of the research.

## 2. Results

### 2.1. Maize Grain Yield Components

**Figure 2:**
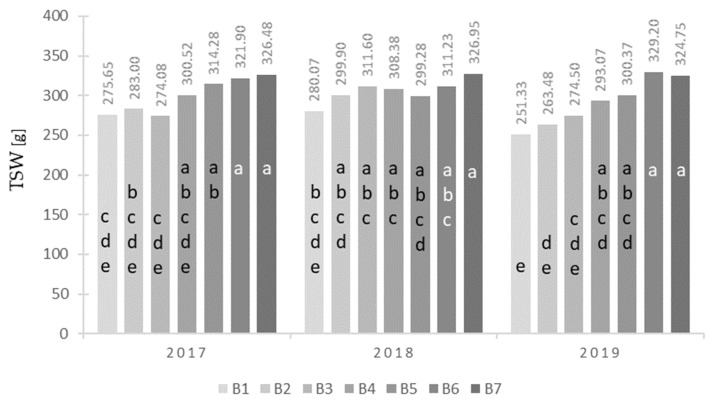
Average TSW [g] values for the combination of years (Y) and fertilizer (B). Values marked with at least one same letter are not significantly different.

The number of production ears per unit area significantly depended on the type of nitrogen fertilizer (Table 1). Significantly, the smallest number of ears per area unit was obtained after the application of urea (B3) and UltraGran stabilo (B7), with the highest obtained by applying urea + N-Lock (B5). Thousand-seed weight (TSW) significantly depended on the type of nitrogen fertilizer (Table 1) and its years of interaction with it (Figure 2). Significantly, the lowest TSW was obtained by maize on the control plot (B1) and ammonium nitrate (B2), and the highest was obtained after the application of the fertilizer UltraGran stabilo (B7). In the first year of the study (2017), the highest TSW was recorded in maize fertilized with urea + N-Lock (B5), Super N-46 (B6), and UltraGran stabilo (B7), while the lowest was recorded for maize fertilized with control (B1) and urea (B3) objects. In 2018, the highest TSW was recorded for the fertilizer UltraGran stabilo (B7), and the lowest was recorded for the control object (B1). In the third year of the study (2019), the highest TSW was recorded for the Super N-46 (B5) and UltraGran stabilo (B7) fertilizers, while the lowest was recorded for the control (B1), ammonium nitrate (B2), and urea (B3) objects (Figure 2). The number of grains per ear depended on the type of nitrogen fertilizer (Table 1). Significantly, the highest value of the assessed features was found in maize fertilized with the Super N-46 (B6) and UltraGran stabilo (B7) fertilizers, while the lowest was found for those fertilized by the control plot (B1) and ammonium nitrate (B2) object.

### 2.2. Grain Yield and Its Moisture Content

**Figure 3:**
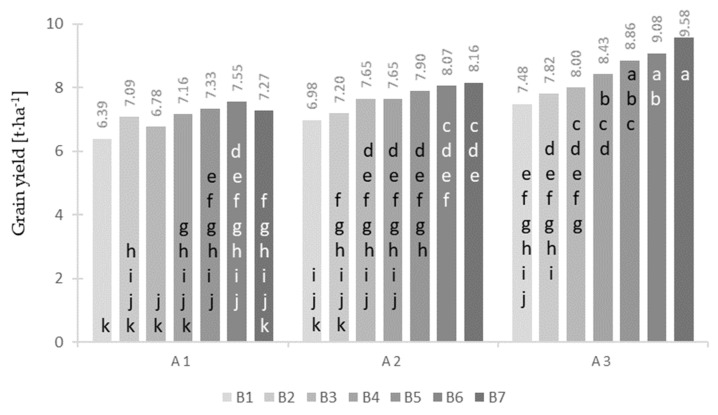
Average grain yield values [t⋅ha^−1^] for the combination of cultivar (A) and fertilizer (B). Values marked with at least one same letter are not significantly different.

Water content in maize grain during harvest significantly depended only on the cultivar (Table 2). The cultivar ES Metronom was characterized by the highest water content in grain compared with the ES Bombastic hybrid. The difference between the examined cultivars was 1.43%. Maize grain yield significantly depended on the study years, cultivar, type of nitrogen fertilizer (Table 2), and interaction of the cultivar with the type of nitrogen fertilizer (Figure 3). Significantly, the highest grain yield was obtained in the first year of the study (2017), and the lowest was obtained in the last year, i.e., 2019. In terms of cultivars, it was found that the ES Metronom hybrid was characterized by the highest yielding potential, while ES Bombastic was significantly the lowest (Table 2). Analyzing the selection of nitrogen fertilizer, it was found that maize produced the lowest grain yield on the control plot (B1), while it produced the highest with nitrogen fertilizers Super N-46 (B6) and UltraGran stabilo (B7) (Table 2). None of the nitrogen fertilizers significantly affected the grain yield of the traditional ES Bombastik hybrid (Figure 3). For the “stay-green” cultivar ES Abakus, the highest grain yield was obtained with the Super N-46 (B6) and UltraGran stabilo (B7) fertilizers, while the lowest was recorded for the control (B1) plot. On the other hand, for the “stay-green + roots power” cultivar ES Metronom, the highest grain yield was recorded for the Super N-46 (B6) and UltraGran stabilo (B7) fertilizers, while the lowest was recorded for the control (B1) plot.

### 2.3. Nitrogen Application Efficiency Indicators

**Figure 4:**
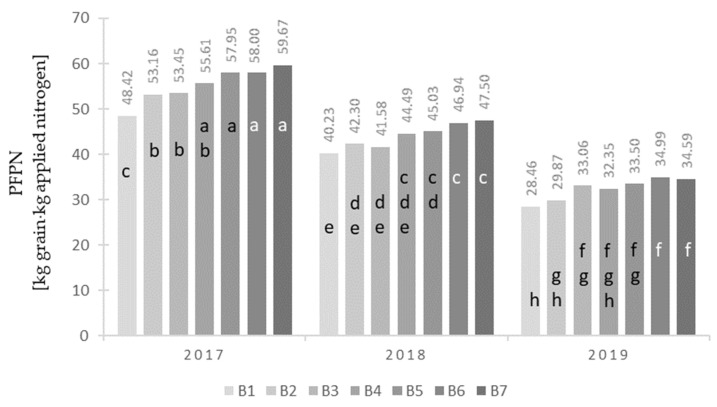
Average PFPN values (kg grain ⋅ kg applied nitrogen) for the combination of years (Y) and fertilizer (B). Values marked with at least one same letter are not significantly different.

The nitrogen content in the maize grain significantly depended on the cultivar and type of nitrogen fertilizer (Table 3). The cultivar ES Metronom was characterized by significantly the highest nitrogen content in grain compared with the cultivar ES Bombastic. Considering the type of nitrogen fertilizer, it was found that the highest nitrogen content in the maize grain was found after applying the fertilizer UltraGran stabilo (B7), and the lowest was found for the control object (B1). Nitrogen uptake with grain yield significantly depended on the years of study, cultivar, type of nitrogen fertilizer (Table 3), and interaction of the cultivar with the type of nitrogen fertilizer (Figure 4). Significantly, the highest nitrogen uptake with grain yield was recorded in the first year of the study (2017), while the lowest yields were recorded in 2018 and 2019. When considering the maize cultivar, we found that the highest nitrogen uptake with grain yield for the cultivar ES Metronom compared with the cultivars ES Bombastic and ES Abakus (Table 3). With respect to the type of nitrogen fertilizer, the highest nitrogen content in the maize grain was recorded after applying the fertilizer UltraGran stabilo (B7), and the lowest was recorded after using the control object (B1). For each of the tested maize cultivars, the lowest nitrogen uptake with grain yield was recorded for the control plot (B1), while the highest was recorded for the plots with nitrogen fertilizers (B6 and B7). It should be noted, however, that the highest values of the examined traits were recorded for the ES Metronom hybrid (Table 4). The partial factor productivity of the applied N fertilizer (PFPN) significantly depended on the study years, cultivar, and type of nitrogen fertilizer (Table 3), as well as the interaction of the cultivar with the type of nitrogen fertilizer (Table 4) and the interaction of the year of research with the type of nitrogen fertilizer (Figure 4). Significantly, the highest increase in grain yield per 1 kg of the applied nitrogen was obtained in the first year of the study (2017), while the lowest was obtained in 2019. When considering the influence of the cultivar on the value of the assessed trait, it was shown that the greatest increase in grain yield per 1 kg of the applied nitrogen fertilizer was obtained for the cultivar ES Metronom, while the smallest was obtained for the cultivar ES Bombastic (Table 3). On the other hand, the lowest value of this feature was found for maize on the control plot (B1), and the highest was found after the application of Super N-46 (B6) and UltraGran stabilo (B7) fertilizers. For each of the tested maize cultivars, the lowest increase in grain yield per kilogram of the applied nitrogen was recorded for the control plot (B1), and the highest was recorded for the plots with nitrogen fertilizers (B6 and B7). It should be noted, however, that the highest values of the examined trait were recorded for the “stay-green + power roots” ES Metronom hybrid (Table 4). The utilization of nitrogen from the nitrogen fertilizer dose significantly depended on the years of study, cultivar, type of nitrogen fertilizer (Table 3), and interaction of the cultivar with the type of nitrogen fertilizer (Figure 4). Significantly, the highest utilization of nitrogen from the dose of nitrogen fertilizer was found in 2017 and 2018 compared with the last year of the study (2019). The cultivar ES Metronom was characterized by the highest nitrogen utilization from the dose of nitrogen fertilizer compared with the cultivars ES Bombastic and ES Abakus (Table 3). Ammonium nitrate (B2) was characterized by the lowest nitrogen utilization from the dose of mineral fertilizer, while UltraGran stabilo (B7) was characterized by the highest. For the traditional hybrid (A1), none of the tested nitrogen fertilizers significantly differentiated the tested feature. In the case of the ES Abakus and ES Metronom hybrids, the lowest nitrogen utilization from the dose of nitrogen fertilizer was recorded for ammonium nitrate (B2), while the highest was recorded for Super N-46 (B6) and UltraGran stabilo (B7).

## 3. Discussion

Weather conditions have a large impact on crop yields, and any deviation from the average meteorological conditions affects agricultural production. Both deficiency and excess of rainfall, as well as too-low or too-high temperature, largely determine the stability and reproducibility of yielding [21]. The years of research varied in weather condition patterns during the maize growing season. The highest grain yield was obtained in 2017, the most favorable year for the growth and development of maize plants. The total rainfall this year was 617 mm, and the average temperature was 13.8 °C. There was no drought recorded in any month (Sielianinov hydrothermal coefficient > 1.0). In 2017, the plants absorbed the most nitrogen and utilized it most optimally. The lowest grain yield was obtained in the dry year of 2019 (total rainfall in the growing season—277 mm). A dry and hot June, as well as a small amount of precipitation in the critical period for maize (July, August), i.e., in the phase of flowering and ear formation, significantly reduced nitrogen uptake and utilization, which translated into the lowest grain yield in the study years. The same relationship was confirmed by many other authors, according to whom precipitation distribution is a factor limiting maize yields in Poland. 

The level of maize yield in the present study depended not only on individual years, but also the experimental factors (cultivar, nitrogen fertilizer). Significantly, the highest grain yield was characteristic of the “stay-green” cultivar ES Metronom (8.46 t⋅ha^−1^). These yields for the cultivars ES Abakus and ES Bombastic were lower by 10% and 19%, respectively. Of all the cultivars tested, the traditional cultivar ES Bombastic generated the lowest yield. The present study showed that the “stay-green” maize cultivars produced a significantly higher grain yield compared with the traditional cultivar. Szulc and Bocianowski [22] demonstrated an identical effect of the cultivar factor on the grain yield. Analyzing the result, they found that the “stay-green” cultivar was characterized by a significantly higher grain yield potential compared with the classic cultivar. This difference amounted to 1.16 t⋅ha^−1^ (the difference in our study was 1.38 t⋅ha^−1^ and 0.8 t⋅ha^−1^). Thus, it can be concluded that the correct selection of a cultivar can be considered one of the most important predictors of maize generative yield. In addition to the cultivar factor, nitrogen fertilization is also a fundamental element affecting the maize grain yield. 

Analyzing individual nitrogen fertilizers, it could be observed that the highest increase in grain yield was recorded in combinations with stabilized fertilizers and urea with the addition of an N-Lock inhibitor. Urea treated with an urease inhibitor showed a lower daily nitrogen loss due to ammonia volatilization [23]. Silva et al. [24] reported a cumulative ammonia loss of 31% for urea and 15% for urea + NBPT in a wide range of soil, weather, and cultivation conditions. In addition, nitrogen preserved in the soil–plant system as a result of the use of urease inhibitors reducing NH_3_ losses contributed to the restoration of soil nitrogen reserves. Slow-release fertilizers, due to their specificity of long nitrogen release, seem to be the best solution for fertilizing “stay-green” maize cultivars. This relationship was confirmed by other scientific reports that implied a fertilization system based on slow-release fertilizers in the cultivation of “stay-green” maize. Higher yields obtained as a result of nitrogen fertilization with stabilized fertilizers resulted from the better uptake and utilization of nitrogen from the applied fertilizers. Water shortages affect nitrogen transformations in the soil. The availability of water in the soil determines the uptake capacity of nitrate ion, which is only mobile in an aqueous environment. Many other authors also indicated the influence of weather conditions on the efficiency of maize fertilization [25,26]. This was consistent with our study, where the highest uptake and utilization of nitrogen from the dose of mineral fertilizer was obtained in 2017. Unfavorable conditions for plant growth were recorded only at the beginning of the growing season during that year (April and the first week of May), while they were favorable during the rest of the growing season. Total precipitation from April to October amounted to 617 mm and was almost twice as high as the sum of precipitation in the years 2007–2019. Judging by the Sielianinov hydrothermal coefficient of water availability, there was no drought in any month of the maize growing season. The lowest uptake and utilization of nitrogen from the dose of mineral fertilizer was obtained in 2019. The latter year was the most unfavorable of the three years of research. Total precipitation during the growing season was only 277 mm. The Sielianiov hydrothermal coefficient of water availability was >1.0 only in May and September. One of the highest water shortages was recorded in June. Very high temperatures during this month additionally intensified the effect of drought. July is the month with the highest average amount of precipitation during the year. The total rainfall for this month in 2019 was only 25 mm. The last such low precipitation measurement at the Experimental Station for the Cultivar Testing in Chrząstowo was recorded in July 2006 (29 mm). According to Księżak et al. [27] and Zielewicz et al. [28], the response of cultivars to nitrogen fertilization could indicate genetic differences in the efficiency of nitrogen utilization. Therefore, the breeding works are aimed at obtaining hybrids with lower nitrogen demands (low-input genotypes). These cultivars are characterized by a better nitrogen utilization capacity, while their ability to transfer this component to the grain is similar to traditional cultivars (high-input hybrids) [29]. Due to the high genotypic variation characterizing maize (*Zea mays* L.), it is possible to find and select certain genotypes that can yield under nitrogen stress conditions [30]. In the present study, the highest amount of nitrogen was taken up by the “stay-green” cultivar ES Metronom. This cultivar was also characterized by the highest nitrogen utilization from the dose of mineral fertilizer. The “stay-green” cultivar, compared with the traditional one, is characterized by a negative indicator of nitrogen remobilization. This means that nitrogen is absorbed from soil resources throughout the growing season (both in the vegetative and generative stages), and soil resources are the main source of nitrogen accumulation in the generative growth stage [13,18]. A statistically lower value in terms of uptake and utilization, as well as nitrogen content, was recorded in the traditional cultivar ES Bombastic, which was characterized by a positive indicator of nitrogen remobilization. The obtained results have implied that the “stay-green” cultivar is able to absorb more nitrogen and utilize it more effectively compared with the traditional cultivar. This tendency was also observed in the second tested cultivar, “stay-green” ES Abakus. It was characterized by a higher nitrogen uptake than the traditional cultivar; however, it was inferior to it in terms of nitrogen utilization (both features statistically insignificant). The statistically comparable variation in the value of this trait for the “stay-green” and traditional cultivars could be attributed to genetic aspects. ES Abakus is a three-way cross hybrid with greater genetic diversity and a lower utilization of the heterosis effect compared with the single-cross ES Bombastic hybrid. Considering the effect of the applied type of nitrogen fertilizer on nitrogen uptake by maize and its utilization, it could be observed that the values describing these characteristics increased along with the duration of the fertilizer action (from B1:B2 to B7). Slower release of nitrogen from fertilizers, due to the action of inhibitors, resulted in a higher efficiency of nitrogen uptake and utilization. The highest nitrogen uptake and utilization was recorded in combination with the stabilized fertilizer UltraGran stabilo, Super N-46, and urea with the nitrification inhibitor N-Lock (statistically significant difference) compared with the control and non-stabilized nitrogen fertilizers (ammonium nitrate and urea). An analogous relationship could be observed in the nitrogen utilization values. Our research showed differences in the uptake and utilization of the nitrogen from different fertilizers by individual cultivars. The traditional cultivar ES Bombastic was characterized by the smallest differences in nitrogen uptake and utilization from the tested nitrogen fertilizers. Although better utilization of nitrogen from stabilized fertilizers compared with conventional fertilizers could be observed, the differences were not significant. This indicated that the traditional cultivar (with a positive nitrogen remobilization coefficient) was not able to fully utilize this nutrient from the stabilized fertilizers. The situation was different for “stay-green” cultivars (with a negative nitrogen remobilization coefficient), namely ES Abacus and ES Metronome. In this case, there were greater differences in the uptake and utilization of nitrogen in favor of stabilized nitrogen fertilizers. The highest significant differences were recorded in the single-cross “roots-power” hybrid, ES Metronom.

## 4. Materials and Methods

### 4.1. Experimental Field

The field experiment was carried out in the years 2017–2019 on the fields of the Experimental Station for the Cultivar Testing in Chrząstowo, belonging to the Research Centre for Cultivar Testing in Słupia Wielka. It was conducted in a split-split-plot design with three experimental factors, in three field replications. The following factors were studied: Y—1st-order factor—years; A—2nd-order factor—maize cultivar: A1—ES Bombastic (FAO 230–240)—single-cross hybrid (SC), A2—ES Abakus (FAO 230–240)—three-way cross hybrid (TC, “stay-green”), A3—ES Metronom (FAO 240)—single hybrid (SC, “stay-green” + roots power). B—3rd-order factor—type of nitrogen fertilizer: B1—control (without N application), B2—ammonium nitrate, B3—urea, B4—ammonium nitrate + N-Lock, B5—urea + N-Lock, B6—Super N-46, B7—UltraGran stabilo. The same level of mineral fertilization was applied in all experimental plots in the amount of 150 kg N ha^−1^, 120 kg P_2_O_5_ ha^−1^, and 130 kg K_2_O ha^−1^. Nitrogen fertilization was not applied in the control combination (B1). Nitrogen fertilizers were applied as broadcast fertilization directly before maize sowing. After application, they were mixed with soil. In combinations with standard nitrogen fertilizers (B4 and B5), N-Lock nitrogen stabilizer was applied as a spray on day 5 after sowing the nitrogen fertilizer at a rate of 1.7 L ha^−1^. It contains 200 g of nitropyrene in the form of a microcapsule suspension and is designed to slow down the nitrification process. Fertilization with P and K was carried out before maize sowing at 2 dates: in the autumn, the previous year (under winter plowing), and in the spring immediately before sowing the maize (before the combined seed drill). At the first date, the compound fertilizer Lubofos 12 (P_2_O_5_—12%, K_2_O—20%) was applied, containing 36 kg⋅ha^−1^ P_2_O_5_ and 60 kg⋅ha^−1^ K_2_O. The remaining dose of P and K was supplemented before maize sowing in the form of enriched superphosphate (40% P_2_O_5_)—84 kg⋅ha^−1^, as well as potassium salt (60% K_2_O)—70 kg⋅ha^−1^.

### 4.2. Determination of Grain Moisture Content and Yield Components

Random samples for moisture content analyses were collected from the threshed mass in each plot. Measurements were taken using a Super Matic electronic hygrometer. Samples collected for moisture analyses were 250 gram in weight. Results are given in percent accurate to two decimal places.

- Number of ears [ears·m^−2^]: all fully formed ears were counted in the two middle rows of each plot. Their number was divided by the size of plot to be harvested;
- Number of kernels in ear [kernels]: the number of kernels in a row and the number of rows were calculated on each of 10 randomly selected ears. The number of kernels in an ear was obtained from the product of these two values;
- 1000 seed weight (TSW) [g]: this value was calculated by adding the results for two randomly collected samples containing 500 seeds each.

### 4.3. Assay Methods

- In the present research, nitrogen content in grain was assessed using the Kjeldahl method with the device Kjeltec^TM^ 2200 FOSS;
- The use of nitrogen per dose of the mineral fertilizer was calculated with the equation:

(1) $$N(\%)=(N_{f}\;-\;N_{c})\times 100/D$$

where:

N—use of nitrogen (%);
N_f_—nitrogen uptake by fertilized plants (kg·ha^−1^);
N_c_—nitrogen uptake by plants in the control (unfertilized) plot (kg·ha^−1^);
D—nitrogen rate (150 kg·ha^−1^).

- Partial factor productivity of fertilizer nitrogen (PFPN) [31]:

(2) PFPN=P/Nr [kg grains·kg nitrogen applied],

where:

P—grain yield;
Nr—nitrogen rate.

- The uptake of nitrogen in the grain yield was calculated with the following formula:

(3) $$\mathrm{Uptake}=\frac{\mathrm{grain}\;\mathrm{yield}\;\times \;\mathrm{content}\;\mathrm{of}\;\mathrm{nutrients}\;\mathrm{in}\;\mathrm{grain}}{150}\;$$

where:

Uptake—in kg·ha^−1^;
Grain yield—in kg·ha^−1^;
Content of nutrients—in %.

### 4.4. Soil Conditions

The analyzed soils of the experimental field belonged to Iva-quality class, a very good rye complex. In terms of grain size, the top horizons of the analyzed soils were classified as loamy sands, and the content of the loam fraction was 4%, silt dust 14%, and sand fraction 83%. The eluvial horizon contained slightly less loam and dust fractions. The enrichment (B) and bedrock levels were definitely more compact. The pH determined in the water extract expressed in pH units was about 7.0, while in KCl it was about 0.5 units lower and was in the upper values of the slightly acidic range. The organic carbon content was approximately 1%, which gives 1.7% humus. The total nitrogen content was 0.086%, and the C:N ratio was about 12:1 (Table 5). The content of assimilable potassium formed was 80.5 mg K⋅kg^−1^, which qualified these soils to the average enrichment class of this element. The amount of assimilable phosphorus and magnesium put the studied soils in a very high abundance class, as the content of these components was: 168.2 mg P⋅kg^−1^, 92.5 mg Mg⋅kg^−1^, respectively (Table 6).

### 4.5. Thermal and Moisture Conditions

In the three-year period (2017–2019), the lowest average daily temperature during the growing season was recorded in 2017 (13.8 °C) (Table 7). Lower average temperatures were recorded in all months than in 2018 and in the 2007–2019 period (except for May and October). The highest average daily temperature during the growing season was recorded in 2018 and was 2.7 °C higher than in 2017. The highest average daily temperatures in the study years were recorded in 2018 in July (20.1 °C) and August (20.9 °C) and in 2019 in June (21.7 °C) and August (20.6 °C). Total precipitation, from April to October 2017 was 617 mm, and it was the highest in the study years, and 242 mm higher than in the 2007–2019 multi-year period (Table 7). The highest amount of precipitation was recorded in July (134 mm) and August (143 mm). The lowest precipitation, both in comparison with 2017 and the multi-year period (2007–2019), was recorded in 2018 (290 mm) and 2019 (277 mm). In 2018, the lowest rainfall during the growing season was recorded in May (5 mm) and August (14 mm), and the highest in July (120 mm). In 2019, the lowest precipitation was recorded in April (monthly sum—3 mm), June (18 mm), and July during the flowering of maize plants (25 mm). The highest monthly precipitation totals were recorded in May and September during the 2019 growing season.

### 4.6. Statistical Analysis

Statistical analyses, including analysis of variance (ANOVA) and the Tukey HSD (honestly significant difference) test for pairwise comparisons of means, were performed separately for the years of the study and for 2017–2019 according to the experimental data models designed as a split-split-plot experiment type [33]. All calculations were performed using Statistica 13.3 and MS Excel. Statistical significance was taken as *p*-value < 0.05.

## 5. Conclusions

Kernel weight (TSW) was the main component of grain yield and was significantly shaped by the interaction of cultivars and nitrogen fertilizers on the nutritional status and physiological indicators of plants at the flowering stage. Maize plants in good nutritional condition at the flowering stage set more kernels in the ear, without a concomitant reduction in TSW. Such properties were demonstrated by the cultivar ES Metronome. This was mainly due to unfavorable weather conditions during grain filling, which occurred in two of the three years of the study. The advantages of the UltraGrain stabilo formulation over ammonium nitrate and urea become visible when a maize cultivar is selected that is capable of efficient nutrient uptake in the pre-flowering period and effective utilization during the grain filling stage (remobilization). The rational fertilization of maize using urea-based nitrogen fertilizer with a urease inhibitor requires the simultaneous selection of cultivars, which are physiologically profiled for efficient nitrogen utilization from this form of fertilizer. The maize cultivar ES Metronom showed a significant advantage over other cultivars under fertilization with UltraGrain stabilo or Super N-46. The interaction of a selectively chosen cultivar with a highly genetically targeted potential for nitrogen uptake from the soil combined with a targeted choice of nitrogen fertilizer is important not only due to production, but also for environmental purposes. The relatively low level of nitrogen utilization from the applied nitrogen fertilizers (<35%) was caused by the high mineral nitrogen content in the soil. Therefore, the effect of individual nitrogen fertilizers depended on their formulation.

## Figures and Tables

**Table 1:** Average values of grain yield components for cultivars (A) and fertilizer (B).

Factors	Levels of Factors	Number of Ear [pcs·m^−2^]	TSW [g]	Number of Grains per Ear [pcs.]
A	A1	8.48 *ns*	287.80 b	402.82 *ns*
	A2	8.55 *ns*	302.37 a	408.39 *ns*
	A3	8.60 *ns*	305.54 a	407.14 *ns*
B	B1	8.51 ab	269.02 e	373.06 c
	B2	8.51 ab	282.13 e	375.65 c
	B3	8.43 b	286.73 de	404.31 abc
	B4	8.51 ab	300.66 cd	399.57 bc
	B5	8.81 a	304.64 bc	403.07 abc
	B6	8.56 ab	320.78 ab	444.58 a
	B7	8.46 b	326.06 a	442.57 ab

Values in columns marked with at least one same letter are not significantly different; *ns*—not significant.

**Table 2:** Average values of grain moisture and yield for years (Y), cultivars (A), and fertilizer (B).

Factors	Levels of Factors	Grain Moisture [%]	Grain Yield [t⋅ha^−1^]
Y	2017	16.47 *ns*	9.73 a
	2018	17.64 *ns*	7.77 b
	2019	15.91 *ns*	5.71 c
A	A1	15.95 b	7.08 c
	A2	16.69 ab	7.66 b
	A3	17.38 a	8.46 a
B	B1	16.83 *ns*	6.95 d
	B2	16.49 *ns*	7.37 cd
	B3	16.51 *ns*	7.48 c
	B4	16.54 *ns*	7.75 bc
	B5	16.58 *ns*	8.03 ab
	B6	17.00 *ns*	8.23 a
	B7	16.74 *ns*	8.34 a

Values in columns marked with at least one same letter are not significantly different; *ns*—not significant.

**Table 3:** Average values of nitrogen utilization efficiency indicators for years (Y), cultivars (A), and fertilizer (B).

Factors	Levels of Factors	Content of N in the Grain [%]	Uptake N [kg·ha^−1^]	PFPN [kg Grains·kg Nitrogen Applied]	Use N [%]
Y	2017	1.67 *ns*	139.16 a	55.18 a	20.03 a
	2018	1.58 *ns*	105.07 b	44.01 b	17.46 a
	2019	1.82 *ns*	88.77 b	32.40 c	11.48 b
A	A1	1.62 b	97.19 b	40.23 c	13.09 b
	A2	1.67 ab	108.39 b	43.37 b	12.78 b
	A3	1.78 a	127.42 a	47.99 a	23.10 a
B	B1	1.55 d	90.01 f	39.04 e	-
	B2	1.66 c	103.41 e	41.78 d	8.93 e
	B3	1.70 bc	108.34 de	42.69 cd	12.22 de
	B4	1.71 abc	112.34 cd	44.15 bc	14.88 cd
	B5	1.72 abc	116.86 bc	45.49 ab	17.90 bc
	B6	1.74 ab	121.19 ab	46.65 a	20.79 ab
	B7	1.77 a	124.85 a	47.26 a	23.23 a

Values in columns marked with at least one same letter are not significantly different; *ns*—not significant.

**Table 4:** Average values of nitrogen application efficiency indicators for the combination of cultivar (A) and fertilizer (B).

A	B	Uptake N [kg·ha^−1^]	PFPN [kg Grains·kg Nitrogen Applied]	Use N [%]
A1	B1	80.36 l	35.83 k	-
	B2	94.13 jkl	40.15 hijk	9.18 fg
	B3	94.60 jkl	38.78 jk	9.49 fg
	B4	100.66 ijk	41.35 ghij	13.54 defg
	B5	101.73 hijk	41.53 fghij	14.24 defg
	B6	105.56 ghijk	42.79 efghij	16.80 cdef
	B7	103.30 ghijk	41.20 ghij	15.30 defg
A2	B1	91.95 kl	39.16 ijk	-
	B2	100.17 ijk	40.89 ghij	5.48 g
	B3	107.35 fghij	43.49 defgh	10.26 efg
	B4	107.64 fghij	43.33 efghi	10.46 efg
	B5	112.79 efghi	44.76 defg	13.89 defg
	B6	117.37 defg	45.71 def	16.94 cdef
	B7	121.43 def	46.27 cde	19.65 bcde
A3	B1	97.72 jk	42.12 efghij	-
	B2	115.93 defgh	44.29 defgh	12.14 defg
	B3	123.07 cde	45.81 def	16.90 cdef
	B4	128.71 bcd	47.77 bcd	20.66 bcd
	B5	136.05 abc	50.19 abc	25.55 abc
	B6	140.65 ab	51.44 ab	28.62 ab
	B7	149.83 a	54.29 a	34.74 a

Values in columns marked with at least one same letter are not significantly different.

**Table 5:** Basic chemical properties of the experimental field soil.

Years	H_2_O	KCl	% N	% C	% Humus	C:N
	pH					
2017	7.01	6.52	0.086	1.037	1.79	12.1
2018	6.96	6.56	0.086	1.037	1.79	12.1
2019	7.07	6.45	0.085	0.987	1.70	11.6

**Table 6:** Macronutrient soil content in the study years.

Years	Phosphorus mg P·kg^−1^	Soil Fertility Class	Content Rating	Potassium mg K·kg^−1^	Soil Fertility Class	Content Rating	Magnesium mg ·kg^−1^	Soil Fertility Class	Content Rating
2017	168.7	I	very high	79.5	III	medium	92.6	I	very high
2018	162.7	I	very high	87.5	III	medium	89.2	I	very high
2019	173.1	I	very high	74.5	III	medium	95.6	I	very high

**Table 7:** Average monthly air temperatures and monthly total precipitation in individual growing season.

Years	IV	V	VI	VII	VIII	IX	X	Sum/Average
Temperatures [°C]								
2017	6.9	15.0	16.8	17.4	18.0	13.0	9.8	13.8
2018	12.4	17.0	18.2	20.1	20.9	16.3	10.6	16.5
2019	9.8	12.1	21.7	18.8	20.6	14.4	10.6	15.4
Many years (2007–2019)	9.0	13.7	17.4	19.1	19.3	13.7	8.6	14.4
Precipitation [mm]								
2017	30	85	62	134	143	64	99	617
2018	49	5	45	120	14	32	25	290
2019	3	72	18	25	44	84	31	277
Many years (2007–2019)	26	56	58	92	60	40	43	375
The Sielianinov hydrothermal coefficient of water availability ^(1)^								
2017	1.4	1.8	1.2	2.5	2.6	1.6	3.2	2.1
2018	1.3	0.1	0.8	1.9	0.2	0.7	0.8	0.8
2019	0.1	1.9	0.3	0.4	0.7	1.9	0.9	0.9
Many years (2007–2019)	1.0	1.3	1.1	1.6	1.0	1.0	1.6	1.2

^(1)^–According to Sielianinov [32].

## Data Availability

Available upon reasonable request.
